# Density-Dependent Mortality of the Human Host in Onchocerciasis: Relationships between Microfilarial Load and Excess Mortality

**DOI:** 10.1371/journal.pntd.0001578

**Published:** 2012-03-27

**Authors:** Martin Walker, Mark P. Little, Karen S. Wagner, Edoh W. Soumbey-Alley, Boakye A. Boatin, María-Gloria Basáñez

**Affiliations:** 1 Department of Infectious Disease Epidemiology, School of Public Health, Faculty of Medicine, Imperial College London, London, United Kingdom; 2 Department of Epidemiology and Biostatistics, School of Public Health, Faculty of Medicine, Imperial College London, London, United Kingdom; 3 Travel and Migrant Health Section, Health Protection Agency Centre for Infections, London, United Kingdom; 4 Health Information Systems, World Health Organization Regional Office for Africa, Brazzaville, Congo; 5 Special Programme for Research and Training in Tropical Diseases, World Health Organization, Geneva, Switzerland; London School of Hygiene & Tropical Medicine, United Kingdom

## Abstract

**Background:**

The parasite Onchocerca volvulus has, until recently, been regarded as the cause of a chronic yet non-fatal condition. Recent analyses, however, have indicated that in addition to blindness, the parasite can also be directly associated with human mortality. Such analyses also suggested that the relationship between microfilarial load and excess mortality might be non-linear. Determining the functional form of such relationship would contribute to quantify the population impact of mass microfilaricidal treatment.

**Methodology/Principal Findings:**

Data from the Onchocerciasis Control Programme in West Africa (OCP) collected from 1974 through 2001 were used to determine functional relationships between microfilarial load and excess mortality of the human host. The goodness-of-fit of three candidate functional forms (a (log-) linear model and two saturating functions) were explored and a saturating (log-) sigmoid function was deemed to be statistically the best fit. The excess mortality associated with microfilarial load was also found to be greater in younger hosts. The attributable mortality risk due to onchocerciasis was estimated to be 5.9%.

**Conclusions/Significance:**

Incorporation of this non-linear functional relationship between microfilarial load and excess mortality into mathematical models for the transmission and control of onchocerciasis will have important implications for our understanding of the population biology of *O. volvulus*, its impact on human populations, the global burden of disease due to onchocerciasis, and the projected benefits of control programmes in both human and economic terms.

## Author Summary

Human onchocerciasis (River Blindness) is a parasitic disease leading to visual impairment including blindness. Blindness may lead to premature death, but infection with the parasite itself (*Onchocerca volvulus*) may also cause excess mortality in sighted individuals. The excess risk of mortality may not be directly (linearly) proportional to the intensity of infection (a measure of how many parasites an individual harbours). We analyze cohort data from the Onchocerciasis Control Programme in West Africa, collected between 1974 and 2001, by fitting a suite of quantitative models (including a ‘null’ model of no relationship between infection intensity and mortality, a (log-) linear function, and two plateauing curves), and choosing the one that is the most statistically adequate. The risk of human mortality initially increases with parasite density but saturates at high densities (following an S-shape curve), and such risk is greater in younger individuals for a given infection intensity. Our results have important repercussions for programmes aiming to control onchocerciasis (in terms of how the benefits of the programme are calculated), for measuring the burden of disease and mortality caused by the infection, and for a better understanding of the processes that govern the density of parasite populations among human hosts.

## Introduction

Human onchocerciasis, also known as ‘river blindness’, is the parasitic infection caused by the filarial nematode *Onchocerca volvulus*. This neglected tropical disease [1] is the second most common cause of infectious blindness worldwide, after trachoma [2], [3]. The parasite is transmitted solely by black fly (*Simulium*) vectors, which breed in fast flowing rivers.

Irreversible unilateral or bilateral blindness is the worst disease sequela of a chronic, cumulative process that was deemed to result from repeated host's inflammatory reactions against degenerating microfilariae in the cornea and triggered by filarial products [4], [5]. More recently, however, it has been proposed that this process is largely due to the endosymbiotic *Wolbachia* bacteria released by dying microfilariae in the anterior chamber of the eye. These bacteria can elicit much of the inflammatory host response that culminates in the lesions characteristic of ocular and dermal onchocercal disease [6] by stimulating the recruitment of neutrophils, the production of chemokines and cytokines, and the release of cytotoxic mediators by neutrophils, which lead to increased corneal opacity, and a range of skin complaints [6], [7], [8]. By contrast, the pathogenesis of retinal lesions may partly arise from autoimmune processes related to cross-reactivity between the *O. volvulus* antigen Ov39 and the human retinal antigen hr44 [9]. Blindness incidence has recently been shown to be associated with past microfilarial load in individuals followed up within the Onchocerciasis Control Programme in West Africa (OCP) cohort [10], confirming the progressive worsening of onchocercal eye disease with parasite exposure.

Although excess human mortality due to onchocercal blindness has been well documented [11], [12], [13], conclusive demonstration of a more direct relationship between parasite load and increased death rate of the human host has proved elusive [14]. New analyses, however, have indicated that, in addition to causing blindness, the parasite can also be directly associated with human mortality [15]. It is possible that this could arise as a result of the parasite exerting immunosuppressive effects on the host both to autologous [16], [17] and heterologous antigens [18], [19] (thus rendering hosts more susceptible to other infections), as well as from systemic effects such as those culminating in epilepsy [20], [21], [22], [23], and growth retardation syndromes [24], [25], among others. Poor nutritional status as a result of loss of visual acuity and blindness has also been implicated in excess mortality [13], [14].

The OCP was launched in West Africa in 1974–1975. Control initially took the form of weekly larviciding of *Simulium damnosum sensu lato* breeding sites (vector control) and surveillance activities in Benin, Burkina Faso, Côte d'Ivoire, Ghana, Mali, Niger and Togo. The programme started at slightly different times in different areas [26]. In 1986 the OCP expanded to include Guinea, Guinea-Bissau, Senegal and Sierra Leone with the aim of protecting the original area from invasion by infected savannah black flies migrating from western and southern locations not covered by the programme. The OCP began mass treatment with the microfilaricidal drug ivermectin (Mectizan®) in selected areas (as a sole measure or in combination with vector control) in 1988, as the main bulk of insecticidal operations in the OCP core area were scaled down 14 years after their commencement [27], [28]. Epidemiological surveillance in the OCP area comprised surveys of vital and clinical status undertaken in sentinel villages, together with an assessment of microfilarial load via skin snips. In many such villages there were repeated surveys. Since the start of the programme until its closure in December 2002, more than 2,000 villages were surveyed in the 11 West African countries finally included in the OCP [15].

In this article we examine the functional relationship between human mortality rate and skin microfilarial density, using the whole of the OCP dataset (spanning from 1974 to 2001, the latter being the year final epidemiological surveys were conducted). Only recently, and using the full OCP dataset, was an association demonstrated between microfilarial load and excess mortality (an increased mortality rate incurred by individuals infected with *O. volvulus* microfilariae compared to uninfected individuals) for both sexes [15]. A positive association between microfilarial load and mortality of the human host had been demonstrated prior to this for males (with more than 100 microfilariae per skin snip) after adjusting for visual acuity but not for females [14].

Crucial to the estimation of the effect of infection is the shape of the relationship between microfilarial load and excess mortality. It is well recognized that measurement error can alter substantially the shape of this relationship and hence the derived population risk estimates [29]. Regression calibration is an approximate method of adjusting for such errors in non-linear relationships which gives reasonably adjusted point estimates of model parameters [30]. This method has been much applied in ionizing radiation epidemiology, as for example in analyses of the Japanese A-bomb survivor data and other radiation-exposed groups [31], [32], [33], [34].

Knowledge of the precise shape of the relationship between parasite load and excess human mortality will be important for its incorporation into mathematical models of transmission dynamics and control [35], [36]. Not only will this have implications for our understanding of the mechanisms regulating parasite abundance in human populations, and our ability to quantify the population impact of mass microfilaricidal treatment, but also for informing policy, as the benefits of onchocerciasis control programmes may be measured not only in terms of blindness cases averted, but also in terms of number of deaths prevented [37]. This will be crucial for the ongoing reassessment of the global burden of disease due to neglected tropical diseases in general [38], [39] and onchocerciasis in particular.

## Methods

### Epidemiological Methods

The methods used in the epidemiological surveys of the OCP have been previously described [14], [40]. At each survey a complete census of the village was conducted, and approximately 84% of persons enumerated in the census were examined [41]. The countries participating in the OCP signed a memorandum of agreement that covered all issues pertaining to the operations and covered clearance for epidemiological, parasitological, and ophthalmological surveys, etc. Our study satisfied the requirements for ethical clearance within the memorandum. Additionally, a committee consisting of the Chief of Units of OCP ensured that the plans and the methodology of work were correctly followed by the technicians in the field. Communities were free to participate in the taking of skin snip samples.

Individuals were only included in the analysis cohort if they satisfied a number of consistency checks (such as consistency of ages between surveys; known sex; consistency of blindness and vital status codes; and correct temporal sequence of registration, examination, and blindness codes), and had been included in at least two surveys (in the last of which they could have been declared dead).

### Microfilarial Load

Parasitological assessment comprised taking bloodless skin snips (with a 2 mm Holth-type corneoscleral punch), one from the right and one from the left iliac crests. The skin snips were placed in distilled water for 30 minutes and any microfilariae that emerged were counted under a dissection microscope. Negative snips were re-incubated up to a total of 24 hours in saline solution. Biopsies taken with this punch are relatively similar in size, weighing between 1 and 3 mg [42]. The microfilarial *load* of each person was measured as the arithmetic mean of the two skin snip *counts*. To estimate microfilarial load at any time linear interpolation was performed between point measurements following the methodology of Little et al. [10], [15]. Microfilarial load was assumed to increase linearly with age, from 0 at age 0; and to vary linearly between measurements but to be constant after the last measurement in a person (Figure S1).

Microfilarial load was lagged under the assumption that any association between parasite load and host mortality would probably be due to an individual's previous microfilarial load rather than their current burden. Two years was the chosen lag because our previous analysis had found the regression coefficient for the relation between host mortality and blindness prevalence to be greatest when a latency of two years was assumed. However, changing lag periods within a 0–4 year range had little impact on the results [10], [15].

Because the OCP used both antivectorial and antimicrofilarial measures, it would be desirable in the analyses to take account of the number of ivermectin treatments received by each person. Unfortunately, the OCP did not keep patient-specific records of drug administration; however, the (therapeutic) coverage of eligible people in villages where treatment with the microfilaricidal drug ivermectin was provided ranged between 85 and 95%. When a separate analysis was conducted of the relationship between microfilarial load and mortality in the period before ivermectin was administered, the results of these analyses were very similar to those for the full dataset [15]. Table 1 summarizes the data used in the current analysis.

**Table 1:** Summary of mortality data collected by the onchocerciasis control programme in West Africa From 1974 through 2001.

Data item	Number
Villages	2,315
Individuals in the whole database	754,895
Subjects selected for cohort after application of inclusion criteria^a^	295,909
Person-years of follow-up	2,454,941
Mean length of follow-up in years	8.30
Deaths	23,333
Bilateral losses of sight	567

a Inclusion criteria comprise all the consistency checks described in the text plus participation in at least two surveys.

### Calculation of Person-Years at Risk and Preparation of Dataset

Person-years were calculated for strata defined by age group (16 groups in total: [0–5), [5–10), …, [70–75), [75+]), sex (2 groups, males and females), country of residence (11 groups, each corresponding to an OCP country), calendar year of follow up (27 groups in total: 1974, 1975, …, 2001), calendar year of first survey (6 groups in total: 1973, 1974–1976, 1977–1979, 1980–1983, 1984–1989, 1990–2001), and microfilarial load (12 groups in total: [0–1), [1–2), [2–3), [3–5), [5–10), [10–20), [20–50), [50–100), [100–200), [200–300), [300–400), [400+]). To acquaint the reader with the data, Table S1 presents the number of deaths and person-years of follow-up by (to facilitate visual inspection), a somewhat coarser stratification of microfilarial load and host age. Because Bayesian Markov Chain Monte Carlo (MCMC) model fitting (the method of choice for our purposes) is computationally intensive, it was necessary to reduce the dataset from the 135,138 (data-containing) cells present in the full person-year/case table. Preliminary analyses had suggested that collapsing over calendar year of follow-up made very little difference to parameter estimates although it did increase the degree of extra-Poisson variability (overdispersion) in the number of deaths per stratum and thus increased parameter uncertainties, making our statistical inference more conservative (Protocol S1). A version of the dataset was prepared collapsing over calendar year of follow-up, containing 11,386 cells, and this was used for all further analyses. Table S2 compares the relative risk of mortality associated with each microfilarial load stratum for the full and collapsed datasets.

### Statistical Models

It was assumed that the expected number of deaths in stratum *i*, was given by,
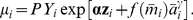
(1)Here *PY_i_* denotes the number of person-years in stratum *i*; **α** = (*α*_0_, *α*_1_, …, *α*_33_), is a vector of regression coefficients; **z** *_i_* is a vector of covariates (for the strata as defined above plus the average year of follow-up and prevalence of blindness in each stratum); 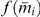 represents the microfilarial dose response, and 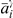 is an interaction term with host age as described below. Following the model presented in Little et al. [15], **z** *_i_* comprises age group, sex, country of residence, calendar year of first survey (as categorical variables), and year of follow-up and prevalence of blindness (as continuous variables). The continuous variables year of follow-up and prevalence of blindness were standardized by subtracting their mean and dividing by their standard deviation. This is standard practice for improving the efficiency of fitting statistical models to data.

The covariate 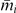 in Equation (1) denotes the average microfilarial load in stratum *i*. The function 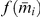 is referred to as the dose-response model (or interchangeably the dose-response function) and, depending on its functional form, it effects either a (log-) linear or non-linear adjustment on the expected number of deaths in stratum *i*. The term 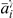 in Equation (1) permits an interaction between microfilarial load and the average age of individuals within a stratum on the expected number of deaths. Average age is denoted 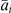 and the parameter *γ* determines the direction and magnitude of the interaction. That is, for a negative value of *γ* and fixed 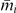, 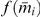 declines with increasing 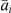 and conversely, for a positive value of *γ* and a fixed 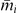, 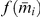 increases with increasing 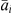. If *γ* = 0 then there is no interaction between age and microfilarial load. The term 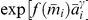 is the relative risk of mortality associated with a mean microfilarial load 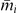 and mean age 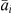. Both 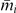 and 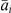 were rescaled by dividing by their respective standard deviations, again to improve the efficiency of fitting the model to the data.

The model presented in Little et al. [15] assumed that the dose-response function was log-linear (and interactions with age were not considered). In this paper, different functional forms were explored, each nested within a sigmoidal-type relationship given by,
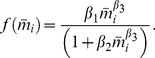
(2)Setting *β*_3_ = 1 yields a hyperbolic functional form which can be linearized under the constraints *β*_3_ = 1 and *β*_2_ = 0. The null model (no relationship between a stratum's mean microfilarial load and the expected number of deaths, corresponding to density independence) is obtained by setting *β*_1_ = 0.

It is noteworthy that although we refer to linear, hyperbolic and sigmoid dose-response models, strictly speaking, these are actually log-linear, log-hyperbolic and log-sigmoid dose-responses (since 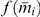 appears in the exponent of Equation (1)) but for the sake of brevity we have dropped this prefix in what follows. This approach (in contrast with models fitted with different forms of 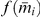 as a multiplicative term outside the exponent of Equation (1)), considerably aided MCMC convergence, yielding much more reliable results, and without compromising the flexibility of the functional form to provide an adequate description of the data. For the reader's convenience, Table 2 lists the definitions of all parameters and variables referred to throughout the text.

**Table 2:** Definitions of parameters and variables.

Parameter/variable	Definition
Dependent variables	
*μ_i_*	Expected number of deaths in stratum *i*
*μ_i_*_0_	Expected number of deaths in stratum *i* not due to infection with (microfilariae of) *Onchocerca volvulus*
Explanatory variables	
*PY_i_*	Number of person-years contributed by all individuals in stratum *i*
**z** *_i_*	A vector indicating the category of age group, sex, country of residence, and year of first survey, and the mean year of follow-up and prevalence of blindness for individuals in stratum *i*
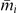	Mean microfilarial load of individuals in stratum *i*
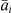	Mean age of individuals in stratum *i*
Estimated parameters	
**α** = (*α*_0_, *α*_1_, …, *α*_33_)	Regression coefficients of the variables comprising **z** *_i_*
**β** = (*β*_1_, *β*_2,_ *β*_3_)	Parameters of the dose-response function given by Equation (2)
*γ*	Parameter of the dose-response age interaction shown in Equation (1)
Fixed parameters	
*k*	Inverse measure of the degree of measurement error, such that the minimum measurement error occurs when *k*→∞, which corresponds to Poisson error. The impact of three values of measurement error were explored; *k*→∞, *k* = 15 (motivated by published data), and *k* = 1 (arbitrarily large)

### Measurement Error

A regression calibration technique [30], similar to that used in analyses of the effects of ionizing radiation on human mortality [31], [32], [33], [34], was used to explore the effect of measurement error in microfilarial loads on the functional form of the dose-response model. Of particular interest was whether measurement error could alter the most parsimonious yet adequate choice of dose-response. The essence of regression calibration is to replace the observed value of a covariate measured with error (here the average microfilarial load per stratum) with its expected value (also referred to as the adjusted value). The regression of the number of deaths per stratum on the expected microfilarial loads per stratum then produces approximately unbiased point estimates of the dose-response parameters (i.e. the *β*s in Equation (2)).

A detailed description of the calculation of measurement error-adjusted microfilarial loads is given in Protocol S2 and Figure S2. The most conceptually important stage of this procedure is the formulation of a measurement error model. Measurement error fundamentally occurs at the individual host level; it describes the distribution of microfilarial counts if counts were hypothetically measured repeatedly from the same person. Consequently, it was assumed that the two microfilarial counts measured per individual (a skin snip was taken from the left and right iliac crests, see section *Microfilarial Load*) were negatively binomially distributed with mean *x* and ‘overdispersion’ parameter *k*. Parameter *k* is an inverse measure of the degree of extra-Poisson variability in microfilarial counts such that as *k*→∞ the distribution becomes Poisson. In this case, it can also be thought of as an inverse measure of the degree of measurement error. Since each individual in the OCP dataset was skin snipped only twice at each follow-up, measurement error could not be reliably estimated from the available data. Consequently, regression calibration was performed by imputing into the statistical model (Equation (1)) measurement error-adjusted microfilarial loads that were calculated assuming the following degrees of measurement error:

1. *k*→∞, corresponding to Poisson measurement error;
2. *k* = 15, corresponding to the value of *k* that was estimated using a published dataset [43] from a previous study that examined 20 repeated microfilarial counts (Figure S3), measured from the left and right iliac crests by skin biopsy in the same way as the OCP data were collected, from each of 15 individuals over a 24-hour period (for details see Protocol S3 and Figure S4);
3. *k* = 1, corresponding to an arbitrarily high degree of measurement error.

### Fitting to Data

Models were fitted with a linear, a hyperbolic or a sigmoid dose-response function (Equation (2)) and with or without an age interaction (i.e. either letting *γ* be estimated or setting it equal to 0). The null model (parasite density-independent human mortality) was also fitted to the data.

Bayesian MCMC techniques [44] were used to fit the models in OpenBUGS [45] (http://www.openbugs.info/w/), the currently maintained and updated version of WinBUGS [46]. Negative binomial errors were assumed for the numbers of deaths in each cell. This form of error structure was used because of evidence of overdispersion (extra-Poisson variability, see Protocol S1). Each parameter was assigned a vague prior, e.g., a normal distribution with mean = 0.0 and variance = 1000 or for the overdispersion parameter of the negative binomial distribution (which must be positive), a gamma distribution with shape and scale parameters = 0.001. Following techniques suggested by Gelman and Rubin [47], three starting values for the Gibbs sampling algorithm were assigned in order to assess convergence on the parameter posterior distributions and to check that our conclusions were not sensitive to the choice of starting values. In general, the first 2,000 samples from each chain were discarded as ‘burn-in’ and a further 4,000 samples were used to estimate the marginal posterior distributions.

### Model Selection

For models fitted to the unadjusted microfilarial loads, goodness of fit was assessed using the deviance information criterion (DIC) [48]. The DIC is a Bayesian generalization of Akaike's information criterion [49], based on a trade-off between the fit of the model to the data and its complexity (number of parameters). The DIC is used to choose between competing models in an analogous way to the AIC [50]; a more complex albeit ‘less parsimonious’ model (a model with more estimated parameters) may be chosen as the most adequate model if it has a lower DIC value compared to a simpler model [48]. The DIC was not an appropriate tool to select among models fitted to measurement error-adjusted microfilarial loads. While the point parameters estimates obtained from models fitted to measurement error-adjusted data are approximately (or sometimes exactly) unbiased, they do not take into account the uncertainty introduced by the adjustment procedure (i.e. the adjusted values are estimates with associated uncertainties) [30], [51]. Consequently, the uncertainty in the dose-response parameters estimated using adjusted microfilarial loads will be underestimated which also renders the DIC invalid. This is because a component of the DIC evaluates the mean deviance (−2×log-likelihood) over the joint posterior distribution of the parameters and, since the dose-response parameters' contribution to the posterior will be inaccurate, potentially spurious DIC values may arise. As a solution to this, log-likelihoods were calculated at the posterior means of the fitted parameters. Since vague priors were used, parameter posterior means are equivalent to frequentist maximum likelihood estimates (MLEs) [44] and therefore the log-likelihood at these values is the model's maximum log-likelihood (MLL). The MLLs were used to conduct pairwise likelihood ratio tests (LRTs) [52] in order to select the most parsimonious yet adequate model.

### Attributable Mortality Risk

The risk of mortality attributable to infection with *O. volvulus* (as assessed by the presence of skin microfilariae) over and above that due to blindness was calculated from each fitted model (Equation (1)) by first calculating for each (microfilaria positive or microfilaria negative) stratum the expected number of deaths not due to infection with *O. volvulus*, i.e., assuming a microfilarial load of zero,
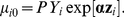
(3)Summing these values over all strata and subtracting from the total number of recorded deaths, *N*, yields the number of deaths attributable to infection with *O. volvulus* (after having adjusted for the prevalence of blindness as this already is a covariate in the models of Equation (1)). The attributable risk of mortality is this value expressed as a percentage,
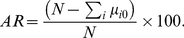
(4)


## Results

Table 3 presents values of parameter estimates (posterior means), DIC, MLL and attributable risk (AR) of mortality due to infection with *O. volvulus* microfilariae for different dose-response models fitted assuming microfilarial loads were measured without error. These are referred to as ‘naïve’ fits. Table 3 also contains the DIC and MLL values for the null model of no association between microfilarial load and human mortality (density independence). Bearing in mind that the lower the DIC the better the fit, the results presented in Table 3 indicate that: a) infection with *O. volvulus* is associated with human mortality, confirming previously published results [15]; b) microfilarial load and age are interactively associated with human excess mortality such that for a given microfilarial load the relative risk of mortality declines with increasing age (indicated by the statistically significantly negative value of parameter *γ*), and c) the sigmoid dose-response relationship was the best fit of those considered suggesting that the relative risk of mortality increases non-linearly initially and saturates at high microfilarial loads. Pairwise comparisons of model MLLs using LRTs confirm the above results with each LRT giving a *P* value<0.001.

**Table 3:** Parameter estimates, maximum log-likelihood, deviance information criterion and mortality risk attributable to *Onchocerca volvulus* infection.

Dose-response model^a^	Age interaction	Posterior means (±95% BCI)				MLL^b^	DIC	AR (%)
		*β*_1_	*β*_2_	*β*_3_	*γ*			
Null	N/A	0^c^	0^c^	1^c^	0^c^	−11,360	22,800	N/A
(Log-)Linear	Yes	0.41 (0.35, 0.45)	0^c^	1^c^	−0.57 (−0.70, −0.42)	−11,235	22,540	5.3
	No	0.26 (0.23, 0.29)	0^c^	1^c^	0^c^	−11,255	22,580	5.0
(Log-)Hyperbolic	Yes	0.73 (0.58, 0.91)	0.29 (0.15, 0.49)	1^c^	−0.55 (−0.68, −0.41)	−11,220	22,510	7.3
	No	0.51 (0.38, 0.51)	0.38 (0.18, 0.36)	1^c^	0^c^	−11,240	22,550	6.7
(Log-)Sigmoid	Yes	1.8 (1.2, 2.8)	1.8 (1.0, 3.1)	2.5 (1.8, 3.5)	−0.59 (−0.72, −0.45)	−11,210	22,490	5.9
	No	1.3 (0.72, 2.42)	2.2 (0.90, 4.6)	2.4 (1.6, 3.6)	0^c^	−11,230	22,530	5.9

a Dose-response models were fitted assuming microfilarial loads were measured without error. BCI: Bayesian Credible Interval; MLL: maximum log-likelihood; DIC: Deviance Information Criterion; AR: Attributable risk; N/A: not applicable.

b Evaluated at the posterior means of the estimated parameters; analogous to the maximum log-likelihood of the model.

c Parameter value fixed, not estimated.

**Figure 1:**
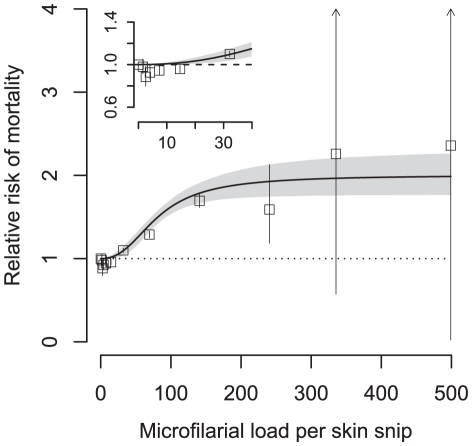
Relative mortality risk with increasing *Onchocerca volvulus* microfilarial load in the Onchocerciasis Control Programme cohort. Observed (open squares) and fitted (solid line) relative risk of mortality, with fitted (log-)sigmoid dose-response model adjusted to the average age of individuals within the cohort. Shaded (grey) area represents the 95% Bayesian credible interval around the fitted line; vertical error bars are 95% confidence intervals around observations. Inset permits visual inspection of the mortality relative risk at parasite loads ≤40 microfilariae per skin snip.

**Figure 2:**
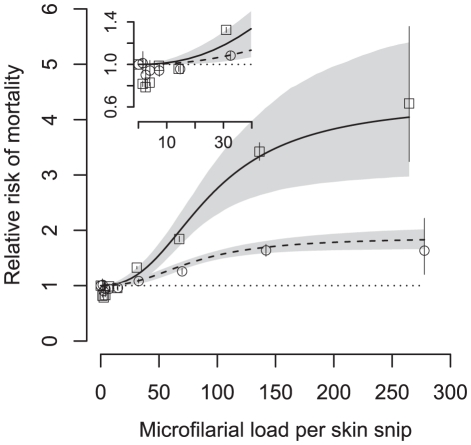
Observed and fitted relative mortality risk with *Onchocerca volvulus* microfilarial skin load according to age-group. Individuals <20 years old (open squares and solid line respectively); individuals ≥20 years old (open circles and dashed line respectively). The fitted sigmoid dose-response model is adjusted to the average age of the respective age groups. Shaded (grey) areas around the fitted lines represent 95% Bayesian credible intervals; error bars represent 95% confidence intervals around observations. Inset permits visual inspection of the mortality relative risk at parasite loads ≤40 microfilariae per skin snip.

The fitted relationship between the relative risk of mortality and microfilarial load derived from the sigmoid dose-response with age interaction is depicted in Figure 1. To aid visual inspection of the fit, model-derived (Protocol S1) point estimates of mortality relative risks are also displayed. To illustrate graphically how the age interaction modifies the relative risk in different age groups, Figure 2 depicts the fitted relative risks (again from the sigmoid dose-response model) for individuals <20 years and individuals ≥20 years. From this figure it is clear that for a given microfilarial load the relative risk of mortality is much greater in the younger age group.

The AR of 5.0% calculated from the linear dose-response model without an age interaction (Table 3) is similar to the 5.2% previously calculated from the OCP data [15]. This is the lowest value of AR, calculated from the poorest fitting model. From Table 3 it is clear that non-linearity in the dose-response leads to higher estimates of AR as does the interaction with host age (with the exception of the sigmoid dose-response model). The AR calculated from the best fit sigmoid dose-response which includes an interaction with host age is 5.9%. Therefore, of the total number of 23,333 (any cause) deaths recorded in the OCP cohort (Table 1), 1,377 would be attributable to infection with *O. volvulus* (in addition to those due by (any cause) blindness).

**Figure 3:**
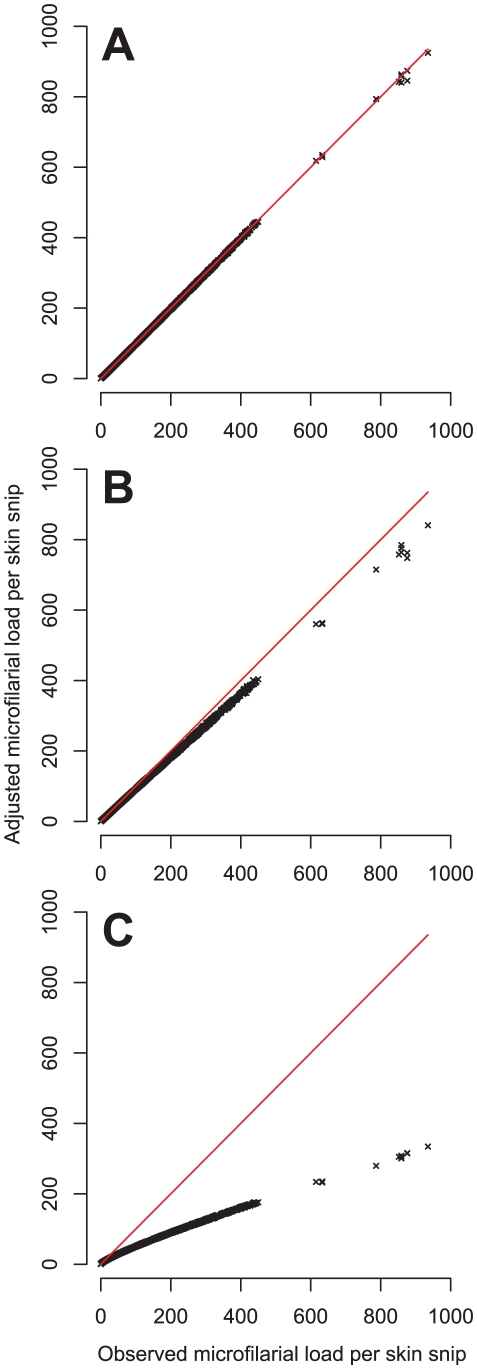
Measurement error-adjusted microfilarial loads plotted against their observed values. Each panel corresponds to an adjustment for a different assumed magnitude of measurement error as defined by parameter *k* of the negative binomial measurement error model (see *Measurement Error* section in the main text). In panel A, *k*→∞ which corresponds to Poisson measurement error. In panel B, *k* = 15 as estimated from published data [43] (see Protocol S3). In panel C, *k* = 1, which corresponds to an arbitrarily large degree of measurement error. In each panel the solid red line is the diagonal representing perfect agreement between observed and adjusted microfilarial loads.

**Figure 4:**
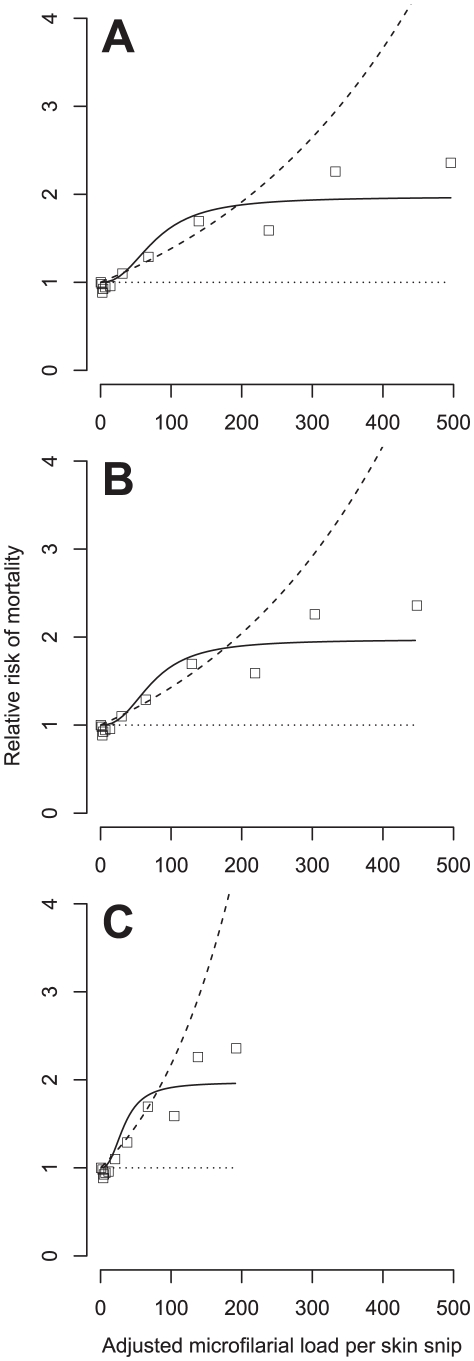
Measurement error-adjusted and fitted relative mortality risk in the Onchocerciasis Control Programme cohort. Panels from top to bottom correspond to adjustments for increasing assumed magnitudes of measurement error as defined by parameter *k* of the negative binomial measurement error model (see *Measurement Error* section in the main text). In panel A, *k*→∞, corresponding to Poisson measurement error. In panel B, *k* = 15, as estimated from published data [43] (Protocol S3). In panel C, *k* = 1. In all panels the solid and dashed lines represent, respectively, the fitted (log-)sigmoid and (log-)linear dose-response models. Note the absence of error bars around the model-derived point estimates of relative risk (Protocol S1) and around the fitted dose-response. This is because regression calibration cannot account fully for the uncertainty introduced by adjusting the observed data for measurement error (see Discussion).

From Figure 3 it can be seen that adjusting microfilarial loads for measurement error decreases the magnitude of large observations and, to a lesser extent, increases the magnitude of small observations. The severity of this effect increases with an increasing magnitude of measurement error. How this affects the fitted dose-response models and the corresponding estimates of AR is shown by the results presented in Table 4, Table 5 and Figure 4.

**Table 4:** Adjusted point parameter estimates, maximum log-likelihoods, and risk of mortality attributable to *Onchocerca volvulus* infection.

Dose-response model^a^	Measurement error^b^	Point estimate (posterior mean)				MLL^c^	AR (%)
		*β*_1_	*β*_2_	*β*_3_	*γ*		
(Log-)Linear	Naïve^d^	0.41	0^h^	1^h^	−0.57	−11,235	5.3
	*k*→∞^e^	0.41	0^h^	1^h^	−0.57	−11,235	5.3
	*k* = 15^f^	0.41	0^h^	1^h^	−0.56	−11,235	5.5
	*k* = 1^g^	0.39	0^h^	1^h^	−0.53	−11,230	7.3
(Log-)Hyperbolic	Naïve	0.73	0.29	1^h^	−0.55	−11,220	7.3
	*k*→∞	0.74	0.30	1^h^	−0.55	−11,215	7.3
	*k* = 15	0.71	0.28	1^h^	−0.54	−11,220	7.5
	*k* = 1	0.50	0.11	1^h^	−0.51	−11,225	8.7
(Log-)Sigmoid	Naïve	1.8	1.8	2.5	−0.59	−11,210	5.9
	*k*→∞	1.8	1.8	2.4	−0.59	−11,210	5.8
	*k* = 15	1.7	1.7	2.5	−0.59	−11,210	5.8
	*k* = 1	0.83	0.79	2.9	−0.59	−11,205	5.8

a Dose-response models were fitted assuming microfilarial loads were measured with error. MLL: Maximum Log Likelihood; AR: Attributable Risk.

b The degree of measurement error is summarized by parameter *k* of the negative binomial distribution, which is an inverse measure of extra-Poisson variation in the distribution of microfilarial counts measured per individual.

c Evaluated at the posterior means of the estimated parameters; analogous to the maximum log-likelihood of the model.

d Naïve: Point parameter estimates assuming no measurement error in microfilarial loads (see Table 3);

e Poisson measurement errors;

f Measurement error estimated from published data [43];

g Arbitrarily large measurement error.

h Parameter value fixed, not estimated.

**Table 5:** Pairwise likelihood-ratio test chi-square values comparing the fit of dose-response relationships.

Pairwise Test	DF^a^	Measurement error^b^					
		*k*→∞^c^		*k* = 15^d^		*k* = 1^e^	
		*χ* ^2^	*P* value	*χ* ^2^	*P* value	*χ* ^2^	*P* value
Linear vs. Null	1	250	<0.001	250	<0.001	260	<0.001
Hyperbolic vs. Null	2	290	<0.001	280	<0.001	270	<0.001
Hyperbolic vs. Linear	1	40	<0.001	30	<0.001	10	0.002
Sigmoid vs. Null	3	300	<0.001	300	<0.001	310	<0.001
Sigmoid vs. Linear	2	50	<0.001	50	<0.001	50	<0.001
Sigmoid vs. Hyperbolic	1	10	0.002	20	<0.001	40	<0.001

a DF: Degrees of freedom.

b The degree of measurement error is summarized by parameter *k* of the negative binomial distribution, which is an inverse measure of extra-Poisson variation in the distribution of microfilarial counts measured per individual.

c Poisson measurement errors.

d Measurement error estimated from published data [43].

e Arbitrarily large measurement error.

Table 4 presents parameter estimates, MLLs and ARs calculated from dose-response models fitted to measurement error-adjusted microfilarial loads. As with the naïve fits, inclusion of an interaction between the dose-response and age improved the fit of all models and consequently, the results presented in Table 4 are from models which included this interaction. Within Table 5 are the results of pairwise LRTs comparing the fit of the dose-response relationships fitted assuming the same magnitude of measurement error. The results in Tables 4 and 5 indicate that: a) the sigmoid dose-response (interacting with host age) is the best fit even at high degrees of measurement error, and b) the parameters of the dose-response are only appreciably altered at high degrees of measurement error (i.e. for *k* = 1). This latter effect is depicted graphically in Figure 4 which shows the best fit sigmoid dose-response models for each assumed magnitude of measurement error. Also included in the figure are the fitted linear dose-response models which illustrate the so-called ‘gradient attenuation’ effect of measurement error; the measurement error-adjusted gradient becomes increasingly steep for increasing assumed magnitudes of measurement error (compare Figure 4A, which corresponds to adjustment for the minimum Poisson measurement errors, *k*→∞, with Figure 4C, which corresponds to adjustment for a high degree of measurement error, *k* = 1).

## Discussion

The analyses presented here demonstrate that excess human mortality is non-linearly associated with microfilarial load in the area covered by the OCP in West Africa. This is an important finding since, in the past, excess mortality caused by onchocerciasis has generally not been considered to be significant (but see [15]), nor has its functional relationship with *O. volvulus* microfilarial load been statistically ascertained. To our knowledge, this is the first time that the functional form of the relationship between microfilarial load and excess mortality of the human host has been explored in relation to the OCP data.

In the ‘typical’ generalized infection with *O. volvulus*, immunosuppression of Th1 and Th2 responses is thought to occur via a specific T regulatory-1 response and the non-specific involvement of IL-10 [53]. The sigmoid (S-shaped) dose-response model is initially increasing slowly (small changes at the lowest end of microfilarial loads), then more rapidly, and subsequently saturating, so it may be that low microfilarial loads exert a smaller effect which increases with infection intensity but beyond a certain microfilarial load there is little increase in immunosuppression and hence in excess mortality. A model with facilitated parasite establishment due to immunosuppression has been presented to explain age-profiles of worm burden in onchocerciasis, which for savannah settings such as those in the OCP area, tend to saturate or decrease beyond 35–40 years of age [54]. This immunocompromised state of individuals infected with *O. volvulus* could leave them more vulnerable to other, possibly fatal infections [17], [18], [19], [55]. Onchocerciasis may also be involved in neuropathology (in the form of epilepsy), growth retardation, and general debilitation of the host, which may also be dependent on parasite burden such that pathology increases with increasing microfilarial load up to a certain point [20], [21], [22], [23], [24], [25]. More generally in helminthiases, the worms not only cause contemporaneous pathology and disability during the period of active infection, but infection typically also poses the risk of later, irreversible chronic sequelae or even early mortality, arising partly from anti-parasite immune responses that can often cause permanent damage through direct or systemic post-inflammatory effects [56].

The decreasing relative risk of mortality with increasing host age indicated by all of our fitted models is an intriguing result and suggests that the cumulative damage caused by chronic infection with *O. volvulus* may have a lesser effect on mortality than on other sequelae. For instance, infection-induced immuno-suppression may leave children more susceptible to potentially lethal infections compared to adults. Alternatively, interactions between the parasite and the immune system may change over time/age. In schistosomiasis for instance, evidence has been presented for an association between a pubertal hormone and reduced intensities of *Schistosoma japonicum* infection and re-infection [57] which lends support to the hypothesis that developmental changes occurring during adolescence are necessary to build resistance to schistosomiasis [58]. It is possible that similar age-related immunological changes occur in onchocerciasis which could potentially reduce levels or impact of immunosuppression in adults, leaving children relatively more vulnerable to serious infections. It has been shown that children from *O. volvulus*-infected mothers (and these will be the majority in highly endemic areas) have not only a substantially higher risk of becoming infected; but also acquire patent infection earlier in life, and tend to develop higher infection levels. When longitudinally followed up during the OCP vector control activities, their infection also persisted at higher levels [59].

A limitation of the present work is that while the analysis was based on individual data, the start and duration of control measures were recorded mainly on a community or regional basis. The OCP recorded the geographical (percentage of communities) and therapeutic (percentage of eligible individuals in a community) coverage of ivermectin treatments but not usually the number of treatments received by each individual in a cohort. Knowledge of an individual's ivermectin treatments would help to explain microfilarial load measurements. However, one would not expect ivermectin to have a major effect on the functional relationship between microfilarial load and host mortality (the immunity-facilitating effects of treatment reverse immunosuppression only temporarily [60], [61]). Indeed a more likely consequence is that the statistical power to discern the functional form of the dose-response would have been reduced due to ivermectin suppressing and somewhat homogenizing microfilarial loads over the course of the OCP (see Figure 5 in [10]). Additionally, in our previous work, the relationship between microfilarial load and mortality was separately assessed in the period before ivermectin was distributed and the results of these analyses were very similar to those for the full dataset [15].

Another possible weakness is that in order to determine the microfilarial load at any time, we linearly interpolated between measurements, and the microfilarial load was assumed to be constant after the last measurement in an individual. This assumption might not fully reflect the true situation. In the early stages of vector control and follow-up (before ivermectin was introduced) microfilarial loads might have increased after the last survey point, whereas later, microfilarial loads might have decreased as a result of ivermectin treatment. Moreover, this effect may depend on host age (and sex) since microfilarial loads tend to increase throughout childhood and early adulthood before saturating or decreasing (or increasing) from middle age onwards for West African savannah settings [36], [41], [54]. If this is the case then it is possible that our assumption of a constant microfilarial load after the last measurement has underestimated microfilarial loads in children (and women [36]), particularly in pre-ivermectin stages of vector control. Consequently, it is possible that an alternative explanation for the observed interaction between the dose-response and host age is that this may be an artefact arising from this assumption rather than an indicator of an underlying biological mechanism. However, since the average length of follow-up was fairly short (8.30 years, Table 1) and the period of latency between exposure and mortality was assumed to be 2 years, it seems unlikely that our assumptions would have introduced such manifest bias.

The use of mid-interval estimates for mortality may have incurred some inaccuracies in event times. Since the mortality endpoint was only ascertained during surveys, if a person died between surveys, the death was deemed to have occurred midway between them. Because intervals greater than 10 years could elapse between surveys, the times imputed for the mortality events may be significantly in error. However, since such events were relatively infrequent, we do not expect that significant bias would be introduced by the use of such mid-interval estimates.

Measurement error or discrepancy between the actual average skin microfilarial load of an individual and the value recorded can arise in two main ways. First, assuming microfilariae are randomly distributed throughout the skin, one would expect counts from the same individual to be Poisson distributed. However, there is uneven dispersion of microfilariae in the body [43] and typically some clustering is seen [62]. Such aggregation leads to extra-Poisson variation which motivated our choice of the negative binomial measurement error model. Second, the skin snip examination and microfilarial counting procedure is subject to observer variation and the sensitivity of the method, the precise form of which is not known. Also, the OCP protocol, by which snips were further incubated for 24 hours only if negative at 30 minutes, leads to underestimation of microfilarial counts in those snips positive after the first half hour as microfilarial emergence increases with time in the incubation medium [63].

Different degrees of measurement error were explored and our conclusion that the non-linear, saturating (S-shaped) function is the best fit to the data was unaffected. However, the exact relationship between microfilarial load and relative risk of mortality is somewhat blurred by the consideration of measurement error, particularly because measurement errors in microfilarial loads have not been previously estimated and remain largely unknown. In general, increasing magnitudes of measurement error increasingly homogenize the data; large microfilarial loads are reduced, small microfilarial loads are increased. To understand intuitively why this occurs, consider that the variability in the observed data comprises both variability introduced by measurement error and underlying variability in individuals' true microfilarial loads. As one assigns an increasing proportion of this variability to measurement error (by decreasing *k* in our model) the residual variability left for the true microfilarial loads is reduced.

The degrees of measurement error assumed in this analysis were chosen for the following reasons. Poisson measurement error (corresponding to *k*→∞) was deemed a good basic model corresponding to a random distribution of microfilariae within the skin and no other sources of error. The value of *k* = 15 was estimated (Protocol S3) from available data previously published [43] comprising 20 repeated microfilarial counts, measured from the left and right iliac crests by skin biopsy in the same way as the OCP data were collected, from each of 15 individuals over a 24-hour period (Figure S3). To the best of our knowledge this is the most comprehensive data on repeated measurements of microfilarial counts and thus an appropriate dataset from which to guide our choice of measurement error. Although there is no forthcoming reason why measurement error arising from the random sampling of microfilariae within the skin (i.e. discounting error introduced by observers examining the medium in which skin snips are incubated for microfilarial enumeration) will vary significantly among individuals and populations, the small study from which the auxiliary measurement error data were derived clearly does not account for the measurement error which may have been introduced into the OCP dataset by the various technicians analysing the skin snips. This is why the robustness of our results was also assessed using an arbitrarily greater magnitude of measurement error (*k* = 1).

Methods of adjusting for the effects of covariate measurement error are well developed for linear [51] and generalized linear models [30]. By applying such methods, previous studies have adjusted for the effects of measurement error in microfilarial load when exploring how microfilarial load relates to: a) the uptake of microfilariae by the black fly (*Simulium*) vector [64], [65], and b) the development of larvae within the vector [66]. Standard methods of adjustment were not valid for the analysis presented here because of the overdispersion evident in the response variable (the number of deaths per stratum) and the non-linearity of the hyperbolic and sigmoid dose-response functions. Consequently, and motivated by methods used in ionizing radiation epidemiology to address similar problems [31], [32], [33], [34], regression calibration was used to obtain approximately adjusted point estimates of the dose-response parameters. A major limitation of this method is that it does not accurately reflect parameter uncertainties.

The Bayesian MCMC techniques used to fit the models in this analysis were employed because MCMC offers a powerful tool for fitting complex non-linear models to data. Indeed it may seem unusual to fit models using Bayesian methods but adjust for measurement error using frequentist regression calibration. A Bayesian approach to measurement error [67], [68], [69] is an attractive alternative to regression calibration because variability in the posterior distribution of the (dose-response) model parameters reflects all the uncertainty introduced by measurement error. However, applying such methods to dose-response models fitted to stratum-level data presents considerable complications. This is because variability introduced by measurement error on the individual dose (microfilarial load) measurements must be passed to uncertainty of the mean dose in the stratum. A two-stage Bayesian method of achieving this has been developed and applied to dose-response models of the effects of ionizing radiation on human mortality [32], [70] but the development of a similar method for the models presented here is beyond the scope of this paper.

Parasite density-dependent host mortality would remove individuals with heavy microfilarial burdens from their community, which could significantly impact on transmission dynamics. Mortality of the human host has already been somewhat incorporated as a function of microfilarial load in models for the transmission of *O. volvulus* [35], [71] but not using functional forms such as described in this article. Previously, a minimum worm burden was required for eye lesions to occur, and the rate of going blind was related to the number of eye lesions, blind individuals experiencing a differential death rate compared to the non-blind [71]. Our model assumes, instead, that excess mortality depends upon (suitably lagged) microfilarial load (in this paper the lag is two years), rather than upon the number of ocular lesions or the rate of becoming blind. However, it is possible, and biologically more plausible, that mortality at a given age may be related to cumulative microfilarial load, or more generally to a weighted sum of microfilarial load rather than just lagged load. The computer simulation ONCHOSIM model [72] incorporates the probability of going blind as a cumulative function of microfilarial load. When a person becomes blind, their remaining lifespan is projected to decrease [71]. This means that while the model includes excess mortality of the blind, it ignores excess mortality among sighted individuals with heavy microfilarial loads.

The economic analysis of the impact of the OCP has been based on predictions based on ONCHOSIM, and therefore on prevention of blindness [73], [74]. The effect of incorporating excess human mortality in the form of the sigmoid model described here (the preferred non-linear model), would imply that most of the benefits (reductions in morbidity and mortality) would accrue once the infection intensity is very much reduced and maintained at low levels in advanced stages of the control programme, making the economic assessment of longer horizons more cost-effective [75]. It would also imply that controlling the infection in the younger sections of the population might be of greatest public health importance [76]. With a parasite whose life expectancy may exceed 10 years [35], [72], and the prospects of onchocerciasis control/elimination in the post-OCP era mainly relying on the mass distribution of ivermectin [77], [78], the required duration, sustainability, and impact of the intervention is of interest to scientists and policy-makers alike [37]. Mathematical models relating infection intensity and ocular morbidity in terms of loss of visual acuity and blindness incidence will be important to help impact assessment in relevant (savannah) areas of the African Programme for Onchocerciasis Control (APOC). The ongoing assessment of the global burden of the neglected tropical diseases [38], [39] is revising previous estimates based on updated data and methodologies, a better understanding of the relationships between infection, morbidity and mortality, and the impact of large-scale control programmes. For onchocerciasis, and in addition to visual impairment, blindness and troublesome itching (included in the previous disease models [79], [80]), it will be important to consider the relationships here described between microfilarial load and excess human mortality. In addition, the interplay between density-dependent processes (parasite-associated human mortality being one of them) and worm distribution among hosts will influence the regulation of parasite population abundance, the stability of the host-parasite system, the rates of reinfection following cessation of control operations [81], [82], and the rates of spread of any drug resistance that may emerge in large-scale ivermectin-based interventions [83], [84].

## Supporting Information

Protocol S1 **Collapsing strata over calendar year of follow-up.**
(PDF)

Protocol S2 **Adjusting microfilarial loads for measurement error.**
(PDF)

Protocol S3 **Estimating measurement error in microfilarial loads from published data.**
(PDF)

Figure S1 **Use of linear interpolation to calculate the person-years contributed to strata defined by microfilarial load.** Points indicate measured microfilarial loads on an ageing participant born in 1974 and sampled on 5 occasions before the end of the study period in 2001. The black horizontal lines represent the boundaries of strata defined by microfilarial load. Linear interpolation between measurements allows estimation of the person-years contributed to each stratum (red dotted arrows).
(EPS)

Figure S2
**Estimation of parameters of the exposure distribution for different magnitudes of measurement error.** Panel A depicts the observed (squares) and fitted (line) cumulative distribution of microfilarial loads on the natural logarithmic (ln) scale. The fits at each magnitude of measurement error (*k*→∞, *k* = 15, and *k* = 1) are indistinguishable by eye and so are represented by a single solid line. Panel B depicts the corresponding estimated exposure distribution (the marginal distribution of the ‘true’ microfilarial loads) for *k*→∞ (solid line) and *k* = 1 (dotted line). The distribution estimated for *k* = 15 is indistinguishable from that estimated for *k*→∞. The estimated parameters of each exposure distribution (see Protocol S2 for parameter definitions) are as follows: for *k*→∞, *π* = 0.43, 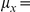
1.86, 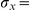
1.61; for *k* = 15, *π* = 0.43, 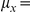
1.88, 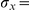
1.60, and for *k* = 1, *π* = 0.43, 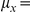
2.13, 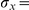
1.41.(EPS)

Figure S3 **Microfilarial loads repeatedly estimated over a 24 h period for 15 patients (data published in** [**43**] **).** The data points in each panel represent microfilarial counts measured from a single individual at 10 different time points in a 24 hour period (at 03:00, 06:00, 08:00,…, 18:00, 21:00, 24:00 hours). At each time point there are 2 microfilarial counts, one from the left and one from the right iliac crests. The thin solid lines link the mean of these 2 counts (the microfilarial load) at successive time points. The thin dashed line links at successive time points the means of the microfilarial loads from all 15 patients. The thick solid and thick dashed lines link the corresponding fitted means from the hierarchical statistical model described in Protocol S3.
(EPS)

Figure S4 **Distribution of 20 microfilarial counts over a 24 h period for 15 patients (** [**43**] **).** The data in each panel are microfilarial counts repeatedly measured from a single individual. The solid line depicts the fitted negative binomial distribution with the maximum likelihood estimate and 95% confidence intervals of the overdispersion parameter *k* displayed on each plot. See Protocol S3 for details.
(EPS)

Table S1 **Number of deaths and person-years of follow-up in the Onchocerciasis Control Programme cohort.**
(PDF)

Table S2 **Mortality relative risk associated with** ***Onchocerca volvulus*** **microfilarial load from full and collapsed OCP datasets.**
(PDF)
