# Supplementary material for: Density-Dependent Mortality of the Human Host in Onchocerciasis: Relationships between Microfilarial Load and Excess Mortality
Source: PLoS Negl Trop Dis. 2012 Mar 27;6(3):e1578. doi: 10.1371/journal.pntd.0001578 (PMC3313942; doi:10.1371/journal.pntd.0001578)
Supplement: Protocol S1 — Collapsing strata over calendar year of follow-up. (PDF) [file pntd.0001578.s001.pdf]

Protocol S1. Collapsing Strata over Calendar Year of Follow-up

The basic structure of the model used to explore the effect of collapsing the original dataset over year of follow-up is given by,

$$\mu_i = PY_i \exp\left[\boldsymbol{\alpha}\mathbf{z}_i + \boldsymbol{\beta}\mathbf{mf}_i\right].$$

Following the main text, μ_i is the expected number of deaths in stratum i, PY_i denotes the number of person-years, $\boldsymbol{\alpha}$ is a vector of regression coefficients and $\mathbf{z}_i$ is vector of covariates comprising age group, sex, country of residence, year of first survey, year of follow-up and prevalence of blindness. The latter was treated either as a categorical or a continuous variable depending on which dataset (full or collapsed) the model was fitted to. In contrast to the model described in the main text, microfilarial load was included as a categorical variable denoted $\mathbf{mf}_i$ (a vector indicating to which microfilarial load group an observation belongs). Correspondingly, $\boldsymbol{\beta}$ is a vector of coefficients (of length 1 minus the number of microfilarial groups) which adjust the expected number of deaths in a stratum for microfilarial load group. As explained in Little et al [1], $\exp(\boldsymbol{\beta})$ gives the relative risk of mortality associated with the different groups of microfilarial load.

The model was fitted to the full 135,138 cell dataset and to the collapsed 11,386 cell dataset. For fitting to the full dataset, calendar year of follow-up was included as a categorical variable with 26 degrees of freedom (corresponding to the 27 groupings). For fitting to the collapsed dataset, the mean calendar year of follow-up (note that collapsing over calendar year of follow-up dismantles the categories of the variable but some adjustment for the variable can still be achieved by including its per stratum mean in the model) was included as a continuous variable.

The model was fitted to the respective datasets using quasi-likelihood techniques which allow for departures from a Poisson error structure [2]. Rather than the variance in the number of deaths per stratum being assumed to be equal to the expected (mean) number of

deaths (Poisson error), the variance was modelled as a linear function of the mean with intercept 0 and gradient θ (a direct measure of dispersion corresponding to the variance-to-mean ratio [3]). For $\theta > 1$ the data are overdispersed relative to the Poisson distribution, the extent of which is quantified by the magnitude of θ.

The fitted relative risks associated with different microfilarial loads are given in Table S2. These values are referred to as 'model-derived' relative risks in the Results of the main text. From these results it is apparent that collapsing over calendar year of follow-up makes very little difference to the estimated relative risk of mortality associated with microfilarial load. This legitimises our approach of collapsing over year of follow-up in order to make the Bayesian fitting procedures outlined in the main text computationally more practical. The estimated confidence intervals are, however, much wider when estimated from the collapsed dataset due to the increased overdispersion in the number of deaths per stratum. Fitted to the full dataset $\theta = 4.17$, indicating a moderate level of overdispersion, while fitted to the collapsed dataset $\theta = 17.7$, indicating an increase in overdispersion. The focus of the analyses presented in the main text was to deduce the functional form of the relationship between excess mortality and microfilarial load (the dose-response). The effect of this increased overdispersion is to increase the likelihood of accepting the null hypothesis of no association between microfilarial load and excess host mortality. Conclusions based on analysis of the collapsed dataset will thus tend to be conservative.

References

1. Little MP, Breitling LP, Basáñez MG, Alley ES, Boatin BA (2004) Association between microfilarial load and excess mortality in onchocerciasis: an epidemiological study. Lancet 363: 1514-1521.

2. McCullagh P, Nelder JA (1989) Generalized Linear Models London: Chapman & Hall.
