# Supplementary material for: Density-Dependent Mortality of the Human Host in Onchocerciasis: Relationships between Microfilarial Load and Excess Mortality
Source: PLoS Negl Trop Dis. 2012 Mar 27;6(3):e1578. doi: 10.1371/journal.pntd.0001578 (PMC3313942; doi:10.1371/journal.pntd.0001578)
Supplement: Protocol S2 — Adjusting microfilarial loads for measurement error. (PDF) [file pntd.0001578.s002.pdf]

# Protocol S2. Adjusting Microfilarial Loads for Measurement Error

Three steps were taken in order to calculate the expected (measurement error adjusted) microfilarial load per stratum. These are summarized below and expounded upon in the subsequent paragraphs:

1. A measurement error model was formulated at the individual host level describing the distribution of microfilarial loads conditional on an unknown 'true' microfilarial load;
2. Bayes' theorem was used to calculate the distribution of the true microfilarial loads conditional on an observed microfilarial load (i.e. the inverse of the measurement error model);
3. The expected value of the true microfilarial load per stratum (the adjusted microfilarial load) was calculated conditional on each of the observed stratum mean microfilarial loads (i.e. for each $\bar{m}_i$).

The measurement error model is briefly described in the main text but to recapitulate, it was assumed that the two microfilarial counts measured per individual (a skin snip was taken from the left and right iliac crests, see section *Microfilarial Load* in the main text) were negatively binomially distributed with mean $x$ and 'overdispersion' parameter $k$. The mean $x$ represents an individual's true but unobserved microfilarial load. Parameter $k$ is an inverse measure of the degree of extra-Poisson variability in microfilarial counts such that as $k \rightarrow \infty$ the distribution becomes Poisson. The observed microfilarial load is the arithmetic mean of two microfilarial counts per individual. This is denoted $m$ (note the distinction between an individual's observed microfilarial load, $m$, and the stratum mean microfilarial load, $\bar{m}_i$).

Because $m$ is the mean of two counts, its distribution is not normal but rather is skewed to the right. A gamma distribution with mean $x$ and variance $x/2 + x^2/(2k)$ was used to approximate this distribution. Here the variance is derived from the variance of the negative binomial

distribution (i.e. the variance of microfilarial counts divided by 2, since 2 microfilarial counts were measured per individual).

The inverse of the measurement error model is the distribution of the true microfilarial loads conditional on an observed microfilarial load. This is found by applying Bayes' theorem,

$$f(x \mid m) = \frac{f(m \mid x) f(x)}{\int_0^\infty f(m \mid x) f(x)\, dx}. \tag{S2.1}$$

Here $f(m \mid x)$ denotes the probability density function (PDF) of the measurement error model (i.e. the PDF of an individual's observed microfilarial load conditional on his / her true microfilarial load) and $f(x)$ denotes the marginal PDF of the true microfilarial loads. The latter is also known as the 'exposure model' [1,2].

The denominator of Equation (S2.1) denotes the (modelled) marginal probability density function (PDF) of the observed microfilarial loads of infected individuals,

$$f(m \mid m > 0) = \int_0^\infty f(m \mid x) f(x)\, dx, \tag{S2.2}$$

where, as described above, $f(m \mid x)$ and $f(x)$ are the PDFs of the measurement error and exposure models respectively. The cumulative distribution function (CDF) of the observed microfilarial loads of infected individuals is,

$$F(m \mid m > 0) = \int_0^\infty F(m \mid x) f(x)\, dx. \tag{S2.3}$$

Some individuals will, of course, be uninfected. Letting $\pi$ denote the proportion of infected individuals, the CDF of the observed microfilarial loads of individuals unconditional on their infection status (infected or uninfected) is,

$$F(m) = (1 - \pi) + \pi \int_0^\infty F(m \mid x) f(x)\, dx. \tag{S2.4}$$

Fixing the magnitude of measurement error (parameter $k$) enabled the parameters of the exposure model and the proportion of infected individuals to be estimated by fitting $F(m)$ to the cumulative distribution of the observed microfilarial load data (i.e. to the cumulative distribution of the $\overline{m}_i$ s). Fitting was achieved my minimising the sum of squares of the differences between the observed and model-predicted cumulative distributions at the average microfilarial load within each microfilarial stratum. Two different distributions were used as candidates for the exposure model; a log-normal and a gamma distribution. The gamma distribution provided a satisfactory fit to the observed data for high assumed degrees of measurement error ($k$ = 1) but was a poor fit for moderate ($k$ =15) and low ($k \rightarrow \infty$) degrees of measurement error. By contrast, the log-normal distribution fitted the data well for all assumed degrees of measurement error (Figure S2) and so was used exclusively in all subsequent calculations. The estimated parameters of the log-normal exposure model (the mean, $\mu_x$, and standard deviation, $\sigma_x$, of the natural logarithm of the true microfilarial loads) in addition to the estimated proportion of infected individuals, $\pi$, are given in the legend of Figure S2.

Having imputed the estimated parameter values of the exposure model (for an assumed degree of measurement error) into Equation (S2.1), the expected values of the stratum microfilarial loads (the measurement error adjusted-microfilarial loads) were approximated by calculating the expected value of $f(x \mid m)$ at each of the observed stratum mean microfilarial loads (i.e. $\mathrm{E}(x \mid \overline{m}_i)$ at each $\overline{m}_i$). The integration necessary to do this was carried out numerically. Each observed $\overline{m}_i$ was then replaced by its measurement error-adjusted (expected) value $\mathrm{E}(x \mid \overline{m}_i)$ in the regression models described in the section *Statistical Models* in the main text.

## References

1. Thomas D, Stram D, Dwyer J (1993) Exposure measurement error: influence on exposure-disease. Relationships and methods of correction. Annu Rev Public Health 14: 69-93.

2. Carroll RJ, Ruppert D, Stefanski LA, Crainiceanu CM (2006) Meansurement error in nonlinear models. London: Chapman and Hall.
