# Supplementary material for: Density-Dependent Mortality of the Human Host in Onchocerciasis: Relationships between Microfilarial Load and Excess Mortality
Source: PLoS Negl Trop Dis. 2012 Mar 27;6(3):e1578. doi: 10.1371/journal.pntd.0001578 (PMC3313942; doi:10.1371/journal.pntd.0001578)
Supplement: Protocol S3 — Estimating measurement error in microfilarial loads from published data. (PDF) [file pntd.0001578.s003.pdf]

Protocol S3. Estimating Measurement Error in Microfilarial Loads from Published Data

The data used to estimate the degree of measurement error in microfilarial load derive from a study conducted by Picq and Jardel [1], working with patients infected with *Onchocerca volvulus* living in the south Sud-Ouest region of Burkina Faso. In this part of Burkina Faso, the savannah species of the *Simulium damnosum* complex predominate, and the activities of the Onchocerciasis Control Programme in West Africa (OCP) began soon after its inception in 1974 [2]. As part of this study, microfilarial counts were measured twice (by skin snip, one from the right and one from the left iliac crests) at 10 time points over a 24 hour period (at 03:00, 06:00, 08:00,..., 18:00, 21:00, 24:00 hours) from each of 15 patients. The 300 microfilarial counts recorded from this procedure are available in the original publication which can be accessed free online at http://www.ncbi.nlm.nih.gov/pmc/articles/PMC2366223/. The data are also plotted in Figure S3.

To recapitulate, the measurement error model described in the main text assumes that microfilarial counts taken from the left and right iliac crests are negatively binomially distributed with mean x and 'overdispersion' parameter k. The mean x represents the 'true' but unobserved microfilarial load and the parameter k is an inverse measure of the degree of measurement error (such that as $k \to \infty$ the measurement error is Poisson). To estimate a common k from the Picq and Jardel data [1], it is necessary to account for different patients having different 'true' microfilarial loads (Figure S3). A crude way of achieving this is to pool the 20 microfilarial counts from each individual (two counts made at 10 time points), estimate k (and the mean, x) for each individual by maximum likelihood (maximum likelihood estimation, MLE) [3], and then calculate an average value of k from the 15 estimates. A graphical summary of the results obtained from performing this procedure is

given in Figure S4. As evident from the asymmetrical confidence intervals displayed on Figure S4, the sampling distribution of *k* tends to be skewed to the right [4], which invalidates the arithmetic mean as a measure of central tendency. However, the inverse of *k*, denoted *c*, has a more symmetrical distribution in addition to other preferable statistical properties [4,5]. For this reason, the estimates and confidence intervals of *k* were actually calculated from MLE of *c*. Similarly, the arithmetic mean of *c* was used to calculate a measure of central tendency for the common *k* which was 7.8 with 95% confidence interval 6.9—9.0.

The method described above for calculating a common *k* was based on the premise that microfilarial counts measured from the same patient at different times during the day are independent. This is a reasonable assumption because the density of *O. volvulus* microfilariae are not thought to exhibit systematic diurnal variation (although see [6,7]). This is clearly evident from Figure S3 which shows that the mean of the microfilarial loads from all 15 patients remained constant over the 24-hour period. However, it is also apparent from Figure S3 that there are considerable random fluctuations in individuals' estimated microfilarial loads over time (that is, fluctuations in the mean of two microfilarial counts measured from the same patient but at different points in time). By not accounting for these fluctuations, the variance attributable to measurement error will be inflated (and the *k* value reduced) and so it is likely that the crude estimate of *k* overestimates the degree of measurement error.

A more efficient and robust way of estimating the *k* common to this dataset is to take a modelling approach, using the data from all individuals to calculate a single *k* while accounting for all likely sources of extraneous variation. To this end, a three-level hierarchical model was constructed, with the hierarchical levels corresponding to: 1) the two microfilarial counts measured from the same individual at the same point in time; 2) the 10

time points at which each patient was skin snipped and, 3) the 15 patients upon which steps 1) and 2) were performed.

At the first and lowest hierarchical level, a microfilarial count j measured at time point i from individual h was assumed to be a realisation from a negative binomial (NB) distribution with mean (the true microfilarial load) x_{ij} and overdispersion parameter (an inverse measure of the degree of measurement error) k,

$$m_{hij} \sim \text{NB}\left(x_{hi}, k\right) \qquad \text{(S3.1)}$$

A linear model was specified at the second hierarchical level, adjusting an individual's true microfilarial load at each time point, $\mathbf{x}_h = (x_{h1}, x_{h2}, ..., x_{h10})$, by a vector of individual-specific regression coefficients, $\boldsymbol{\beta}_h = (\beta_{h1}, \beta_{h2, ...,} \beta_{h10})$,

$$\ln\left(\mathbf{x}_h\right) = \boldsymbol{\beta}_h \mathbf{T}. \qquad \text{(S3.2)}$$

Here $\mathbf{T}$ denotes a 10 × 10 matrix indicting at which time point microfilarial measurements were made. The baseline microfilarial load (β_{h1}) was set at 12:00 so that all other microfilarial loads were adjusted relative to this. At the third hierarchical level, the vector of individual-specific regression coefficients was assumed to be a realisation from a multivariate normal (MVN) distribution,

$$\boldsymbol{\beta}_h \sim \text{MVN}\left(\boldsymbol{\mu}, \boldsymbol{\Sigma}\right), \qquad \text{(S3.3)}$$

where $\boldsymbol{\mu} = (\mu_1, \mu_2, ..., \mu_{10})$, with elements denoting the adjustment at each time point averaged over the 15 individuals. The diagonal entries of the covariance matrix $\boldsymbol{\Sigma}$ represent the variances of the regression coefficients among the 15 individuals and the off-diagonals their covariances.

The model was fitted using Bayesian hierarchical techniques in OpenBUGS [8] (http://www.openbugs.info/w/), the currently maintained and updated version of WinBUGS [9]. The parameters $\boldsymbol{\mu}$ and $\boldsymbol{\Sigma}$ were assigned a conjugate normal-inverse-Wishart distribution [10] such that,

$$\begin{aligned}&\boldsymbol{\Sigma} \sim \text{Inv-Wishart}_{\nu}\left(\boldsymbol{\Omega}^{-1}\right),\\&\boldsymbol{\mu} \mid \boldsymbol{\Sigma} \sim \text{MVN}\left(\boldsymbol{\xi}, \boldsymbol{\Sigma}\right).\end{aligned} \tag{S3.4}$$

Here v and $\boldsymbol{\Omega}$ denote, respectively, the degrees of freedom and the scale matrix of the inverse-Wishart distribution. A typical vague parameterisation was applied by setting $\boldsymbol{\Omega}$ to the identity matrix (diagonals equal to 1, all other entries equal to 0) and v to the order of $\boldsymbol{\Sigma}$ (10 in this case) [11]. The vector of prior means, $\boldsymbol{\xi}$, was set to 0. Three starting values for the Gibbs sampling algorithm were assigned in order to assess convergence on the parameter posterior distributions and to check that convergence was not sensitive to the choice of starting values [10,12]. The first 5,000 samples from each chain were discarded as burn-in and a further 5,000 samples were used to evaluate the posterior distribution.

The posterior mean of parameter k thus estimated was 15 with 95% Bayesian credible interval 11–21. As anticipated, this represents a significantly lower degree of measurement error than was estimated by the crude method. The fit of the model to the data is depicted by the thick lines in Figure S4. Overall, the model appears to describe the data well, accurately capturing the majority of the fluctuations in microfilarial load estimated throughout the day while also fitting well to the mean of the microfilarial loads from the 15 patients at each time point.

References

1. Picq JJ, Jardel JP (1974) [(A method of evaluating microfilaria densities of *Onchocerca volvulus* Leuckart, 1893, in onchoceriasis patients. Assessment of microfilarial densities by site and levels of prevelance in skin biopsies; variations of microfilarial densities over a 24 hour period)]. Bull World Health Organ 51: 145-153 (In French).
2. World Health Organization (1985) Ten years of onchocerciasis control in West Africa. Geneva: World Health Organization. OCP/GVA/85.1B OCP/GVA/85.1B.

3. Bliss CI, Fisher RA (1953) Fitting the negative binomial distribution to biological data - note on the efficient fitting of the negative binomial. Biometrics 9: 176-200.

4. Ross GJS, Preece DA (1985) The negative binomial distribution. Statistician 34: 323-336.

5. Lloyd-Smith JO (2007) Maximum likelihood estimation of the negative binomial dispersion parameter for highly overdispersed data, with applications to infectious diseases. PLoS One 2: e180.

6. Thomas DB, Anderson RI, MacRae AA (1973) Daytime variation in the density of *Onchocerca volvulus* microfilariae in human skin. Bull World Health Organ 49: 493-498.

7. Duke BO, Scheffel PD, Guyon J, Moore PJ (1967) The concentration of *Onchocerca volvulus* microfilariae in skin snips taken over twenty-four hours. Ann Trop Med Parasitol 61: 206-219.

8. Thomas A, O'Hara R, Ligges U, Sturtz S (2006) Making BUGS Open. R News 6: 12-17.

9. Lunn DJ, Thomas A, Best NG, Spiegelhalter DJ (2000) WinBUGS - a Bayesian modelling framework: concepts, structure and extensibility. Stat Comput 10: 325-337.

10. Gelman A, Carlin BR, Stern D, Rubin DB (2004) Bayesian Data Analysis. London: Chapman & Hall.

11. Congdon P (2006) Bayesian Statistical Modelling. New York: John Wiley & Sons.

12. Gelman A, Rubin DB (1992) Inference and iterative simulation using multiple sequences. Statist Sci 7: 457-472.
