# Supplementary material for: Density-Dependent Mortality of the Human Host in Onchocerciasis: Relationships between Microfilarial Load and Excess Mortality
Source: PLoS Negl Trop Dis. 2012 Mar 27;6(3):e1578. doi: 10.1371/journal.pntd.0001578 (PMC3313942; doi:10.1371/journal.pntd.0001578)
Supplement: Table S1 — Number of deaths and person-years of follow-up in the Onchocerciasis Control Programme cohort. (PDF) [file pntd.0001578.s008.pdf]

Table S1. Number of deaths and person-years of follow-up[a] in the Onchocerciasis Control Programme cohort

Microfilarial load per skin snip	Age (years)	Person-years	Deaths
[0, 1.71]	[2.15, 18.1]	367,952.7	3,066
(1.71, 4.46]	[2.15, 18.1]	21,009.7	199
(4.46, 18.1]	[2.15, 18.1]	44,125.7	452
(18.1, 114]	[2.15, 18.1]	57,391.3	815
(114, 935]	[2.15, 18.1]	9,600.0	240
[0, 1.71]	(18.1, 32.6]	379,264.1	3,293
(1.71 ,4.46]	(18.1, 32.6]	20,859.3	176
(4.46, 18.1]	(18.1, 32.6]	45,280.0	436
(18.1, 114]	(18.1, 32.6]	56,521.2	799
(114, 935]	(18.1, 32.6]	9,094.1	210
[0, 1.71]	(32.6, 47.5]	367,854.3	2,915
(1.71, 4.46]	(32.6, 47.5]	20,924.3	179
(4.46, 18.1]	(32.6, 47.5]	44,114.1	460
(18.1, 114]	(32.6, 47.5]	56,135.0	778
(114, 935]	(32.6, 47.5]	9,279.0	209
[0, 1.71]	(47.5, 62.3]	338,410.8	2,645
(1.71, 4.46]	(47.5, 62.3]	21,484.3	179
(4.46, 18.1]	(47.5, 62.3]	43,384.5	441
(18.1, 114]	(47.5, 62.3]	55,921.6	818
(114, 935]	(47.5, 62.3]	9,114.7	225
[0, 1.71]	(62.3, 92.8]	346,566.6	3,113
(1.71, 4.46]	(62.3, 92.8]	21,276.2	208
(4.46, 18.1]	(62.3, 92.8]	43,156.3	449
(18.1, 114]	(62.3, 92.8]	56,727.3	804
(114, 935]	(62.3, 92.8]	9,518.4	224

[a] Stratified by microfilarial load per skin snip and host age. The stratification of microfilarial load and age is based on the variables' quintiles, creating 25 cross-classified groups.
