# Supplementary material for: Density-Dependent Mortality of the Human Host in Onchocerciasis: Relationships between Microfilarial Load and Excess Mortality
Source: PLoS Negl Trop Dis. 2012 Mar 27;6(3):e1578. doi: 10.1371/journal.pntd.0001578 (PMC3313942; doi:10.1371/journal.pntd.0001578)
Supplement: Table S2 — Mortality relative risk associated with Onchocerca volvulus microfilarial load from full and collapsed OCP datasets. (PDF) [file pntd.0001578.s009.pdf]

Table S2. Mortality relative risk associated with *Onchocerca volvulus* microfilarial load from full and collapsed OCP datasets

**Microfilarial load per skin snip**	**Estimated relative risk of mortality (± 95% confidence interval[a])**	
	Fitted to full OCP dataset	Fitted to collapsed OCP dataset
[0,1)	1	1
[1,2]	0.97 (0.82, 1.16)	0.98 (0.68, 1.42)
(2,3]	0.88 (0.71, 1.09)	0.88 (0.56, 1.40)
(3,5]	0.92 (0.77, 1.09)	0.93 (0.64, 1.33)
(5,10]	0.93 (0.81, 1.07)	0.94 (0.71, 1.26)
(10,20]	0.95 (0.84, 1.07)	0.96 (0.74, 1.25)
(20, 50]	1.10 (1.00, 1.21)	1.10 (0.88, 1.37)
(50,100]	1.30 (1.16, 1.45)	1.29 (1.01, 1.65)
(100,200]	1.72 (1.49, 1.97)	1.70 (1.25, 2.30)
(200,300]	1.60 (1.12, 2.30)	1.59 (0.75, 3.39)
(300, 400]	2.36 (1.08, 5.15)	2.26 (0.44, 11.64)
(400, 935]	2.53 (0.61, 10.56)	2.36 (0.12, 47.38)

[a] Confidence intervals were calculated from the estimated standard errors obtained from the Fisher information matrix.
